# Absence of Ledgf in mouse brain affects the Kmt2a/b and polycomb balance, synaptic transmission and motor function

**DOI:** 10.1186/s40478-025-02216-4

**Published:** 2026-01-23

**Authors:** Laura Debusschere, Eduard Bentea, Cecilia Iglesias-Herrero, Nicolas Peredo, Siska Van Belle, Nam Joo Van der Veken, Anna Barber-Janer, Dieter Plessers, Wouter Peelaerts, Wout Hannes, Martine Michiels, Chris Van den Haute, Veerle Baekelandt, Zeger Debyser

**Affiliations:** 1 ADVANTAGE, Department of Pharmacological and Pharmaceutical Sciences, KU Leuven, Leuven, Flanders Belgium; 2 Laboratory for Neurobiology and Gene Therapy, Department of Neurosciences, Leuven Brain Institute, KU Leuven, Leuven, Flanders Belgium; 3 Center for Brain and Disease Research, VIB Bioimaging Core Leuven, VIB Technologies, Leuven, Belgium; 4 Department of Neurosciences, VIB Bioimaging Core Leuven, KU Leuven, Leuven, Belgium; 5 Leuven Viral Vector Core, KU Leuven, Leuven, Belgium

## Abstract

Lens epithelium-derived growth factor (LEDGF), encoded by the *Psip1* gene, exists in two splice variants, LEDGF/p75 and LEDGF/p52. Although little is known about its role in the brain, LEDGF has been proposed to play a role in neurogenesis. Since known LEDGF binding partners, such as PogZ, CDA7L, MLL1 and MeCP2 are implicated in neurological dysfunction, we investigated the role of LEDGF in mouse brain. We developed a conditional *Psip1* knock-out (cKO) mouse model by crossbreeding *Psip1*^fl/fl^ mice with Nestin^Cre^ mice, resulting in neuronal depletion of both isoforms in the central nervous system. In wild-type (WT) animals, brain region-dependent alternative splicing was evidenced, with more p75 over p52 in the cerebellum and more p52 over p75 in the hippocampus. Behavioral phenotyping revealed that already at a young age, *Psip1* cKO mice show motor deficits. In cerebellar neurons, LEDGF depletion results in more and smaller MeCP2 condensates. Bulk and comparative RNA sequencing of cerebellar extracts revealed downregulation of genes involved in synaptic transmission. Moreover, transcription factor network analysis showed that the differentially expressed genes are mainly regulated by the Polycomb repressive complex 2 (PRC2). Since the LEDGF/p75 binding partner MLL1 is part of the Trithorax Complex, the counterpart of PRC2 in gene regulation, our data highlight the importance of LEDGF/p75-mediated regulation of synaptic gene expression in the cerebellum through Trithorax.

## Introduction

Lens epithelium derived growth factor (LEDGF) is encoded by the *PSIP1* gene and exists in 2 isoforms, LEDGF/p75 and its alternative splice variant LEDGF/p52 [1]. LEDGF proteins are members of the hepatoma-derived growth factor (HDGF) family characterized by the presence of a conserved amino-terminal PWWP domain. The PWWP domain reads H3K36 dimethylated and trimethylated marks and thereby recruits LEDGF to the body of actively transcribed genes [2]. The C-terminal portion of LEDGF/p75 harbors the integrase binding domain (IBD), known to recruit cellular proteins to the chromatin [3–5]. Little is known about a potential role of LEDGF in brain function. Chylack et al. found high expression of LEDGF/p75 in the germinal neuroepithelium and cortical plate regions in human fetal brain [6]. High expression levels of LEDGF/p75 in the subventricular zone of adult and aged human brain suggest a persistent role in neurogenesis and differentiation of neuroepithelial stem cells [6].

At least four LEDGF interacting partners have been associated with brain diseases. Both isoforms of LEDGF interact with Methyl-CpG binding protein 2 (MeCP2) via their PWWP-CR1 domain [7–9]. *MECP2* mutations are known to cause Rett syndrome (RTT), a progressive neurodevelopmental disorder that predominantly affects girls [10]. Affected girls develop normally until they are 6–18 months old, when higher brain functions start to decline resulting in loss of speech and purposeful hand movement, ataxia and seizures [11]. Parkinsonian symptoms are often observed in older RTT patients [12, 13]. RTT mouse models, mainly those lacking the *Mecp2* gene, demonstrate behavioral features such as hypoactivity and motor impairments including hindlimb clasping, as well as changes in anxiety and repetitive behavior [14, 15]. Moreover, duplication of the *MECP2* gene is giving rise to the MeCP2 duplication syndrome (MDS) which is predominantly affecting boys. These boys show intellectual disability, developmental delay, hypotonia, recurrent infections, seizures, gastrointestinal problems and dysmorphic features [16]. The Pogo transposable element with zinc finger domain (POGZ), is a LEDGF/p75 specific binding partner [5, 17]. POGZ is a protein involved in chromatin remodeling and gene regulation via its interaction with heterochromatin protein 1 (HP1) [18]. Mutations in the *POGZ* gene are associated with the White-Sutton syndrome, a neurodevelopmental disorder characterized by intellectual disability, microcephaly, hyperactivity and vision problems [18]. *PogZ* deficient mice show microcephaly, growth impairment, increased sociability and learning and motor deficits mimicking several of the human symptoms [18]. The cell division cycle-associated 7-like protein (CDA7L) also known as JPO2 or RAM2, also interacts with the IBD of LEDGF/p75 [5, 19]. CDA7L plays a role in c-Myc-dependent transformation in medulloblastoma [20]. Mixed-lineage leukemia 1 (MLL1), also known as Histone-lysine N-methyltransferase 2A (KMT2A), is another well-known binding partner of the IBD domain of LEDGF/p75 [5, 21]. The interaction of LEDGF/p75 with MLL1 is well studied in the field of leukemia, where LEDGF/p75 is tethering MLL fusions towards *Hox* genes [21]. However, MLL1 has also been implicated in neurogenesis, synaptic plasticity and working memory [22, 23]. De novo mutations in the *MLL1* gene are the cause of the Wiedemann-Steiner syndrome, a rare neurodevelopmental disorder [24]. Patients show hypertrichosis, short stature, intellectual disability and a typical facial appearance [24]. A conditional *Mll1* KO mouse model targeting forebrain neurons is the only available mouse model and exhibits hypoactivity, increased anxiety-like behaviour and memory impairments [22, 25].

Given the role of these binding partners in neurobiology, we hypothesized that LEDGF depletion could impact brain function. Given that a full body knockout (KO) of *Psip1* in mice leads to high perinatal lethality, probably due to the role of LEDGF in controlling *Hox* gene expression during development [26], we used the Cre-LoxP system to induce a conditional *Psip1* KO in the central nervous system. After validation of the KO on protein level by Western Blotting and immunohistochemistry, we determined the behavioral phenotype of the conditional *Psip1* KO model. Expression of known binding partners was verified. Finally, transcriptomics of both hippocampal and cerebellar extracts elucidated how Polycomb Repressive Complex 2 (PRC2) downregulates synaptic gene expression upon depletion of LEDGF in the brain.

## Methods

### Animals

Homozygous *Psip1*^fl/fl^ mice [27] on a C57BL/6 J background were crossed with heterozygous Nestin^Cre/−^ mice [28] on a C57BL/6 J background resulting in heterozygous *Psip1*^*WT/fl*^ Nestin^Cre/−^ or heterozygous *Psip1*^*WT/fl*^ mice (F1 generation). Next, these mice were crossed to obtain homozygous *Psip1*^*fl/fl*^ Nestin^Cre/−^ mice (F2 generation). The resulting *Psip1*^*fl/fl*^ Nestin^Cre/−^ mice, hereafter referred as cKO mice, were crossbred with homozygous *Psip1*^fl/fl^ mice to maintain the KO of *Psip1* in the central nervous system. For the control mice, a separate parallel breeding was kept to maintain the heterozygous state of Nestin^Cre/−^ mice, hereafter referred as wild-type (WT) mice. Mice were group-housed in individually ventilated cages in a room with a 14 h light/10 h dark cycle and temperature of 20–22 °C. They had access to food and water ad libitum. All animal experiments were approved by the KU Leuven Bioethical committee [P200/2019; Belgium] and performed according to the European directive on the protection of animals used for scientific purposes [2010/63/EU].

### Genotyping

An ear clip was taken from mice and genotyping was performed using the KAPA Mouse Genotyping Kit (Roche) according to the manufacturer’s instructions. In the case of the WT allele of the *Psip1* gene, a PCR band is present at 745 bp, while for the loxP allele, a band is present at 802 bp. If the Cre gene is present, a PCR band for Cre of 350 bp is observed. The PCR program for *Psip1* was: 3′ 95 °C, 15″ 95 °C, 15″ 55 °C, 30″ 72 °C, 10′ 72 °C, repeating step 2–4 30 times. The PCR program for Cre was: 3′ 95 °C, 15″ 95 °C, 15″ 58 °C, 30″ 72 °C, 10′ 72 °C, repeating step 2–4 30 times. Primers used for *Psip1* were: sense strand 5’ GAGATATCGAGGCAGAAAGAAGACTGGGATAG 3’; antisense strand 5’ TGGAATTCTATCTCAAACAAACCAAAGAGC 3’. Primers used for Cre were: sense strand 5’ ATTTGCCTGCATTACCGGTC 3’; antisense strand 5’ ATCAACGTTTTCTTTTCGG 3’.

### Behavioral testing

All behavioral tests were performed during the light cycle, between 8:00 and 18:00. Behavioral assays were performed at different ages with male and female mice. To eliminate bias from olfactory cues, each apparatus was cleaned with Umonium 38 Medical Spray (Laboratoire Huckert’s International, Wavre, Belgium) before and after each animal was tested, except for the rotarod which was cleaned with mild detergent. Mice were habituated to the testing room 1 h prior to the experiment.

*Open field test*. The open field test was done in an arena of 50 × 50 cm with a white background to easily detect the mice. The center area was defined as an inner square of 25 × 25 cm. Mice were placed in the center of the arena and their movement was recorded for 10 min using the ANYmaze® software.

*Rotarod*. An accelerating rotarod was used in this experiment, with four mice being tested simultaneously. On the first day, mice were subjected to a training trial on a fixed-speed rod rotating at 4 rotations per minute (RPM) during 60 s, allowing the mice to adapt to the apparatus. After a one-hour rest period, the mice were tested in three trials with a 5-min interval between each trial. On the first day, the rod accelerated from 4 to 40 RPM over 5 min. On the second and third day, the rod accelerated from 4 to 45 RPM over 5 min. Results were measured by recording the duration before falling of the rod. The average of the three trials per day was calculated for analysis. Passive rotation was not permitted and was counted as falling of the rod.

*Elevated Plus Maze*. The elevated plus maze consisted of four elevated arms (50 cm from the floor) arranged in a plus-shape with 2 opposite arms being open (35 × 5 × 0.5 cm) and 2 being closed (35 × 5 × 17 cm). In the open arms, raised edges of 0.5 cm were used to ensure the mice did not fall of the maze during exploration. Mice were placed in the center of the maze facing towards one open arm and were allowed to freely explore the arena for 10 min. Their movement was recorded with ANYmaze® software.

*Pole test*. Mice were placed, with their head facing upwards, at the top of a vertical, rough-surfaced pole (50 cm long). The mice should turn to face downwards and descend into their cage. To ensure the mice descended, the pole was placed in their home cage. The mice were tested for the time necessary to turn downwards (Time to turn) and the total time needed to descend from the pole into their home cage once they turned downwards (Time to descend). The total duration of the pole test was also determined (Total time). On the first day, the mice were allowed to descend the pole without recording for ensuring habituation to the test. On the second day, the test was repeated three times with a 30 s interval between trials. The average of the three trials was taken for further analysis.

*Hindlimb clasping*. Mice were held by their tail and objects in their surroundings were avoided. Three consecutive trials of 10 s were performed for each mouse with a 20 s intertrial interval. Each trial was recorded using a video camera. Afterwards the mice were placed back into their cages. Every video was afterwards assessed manually by a researcher blinded to the genotype. To assess the degree of hindlimb clasping, a score system was used. If the hindlimbs were splayed outwards and away from the abdomen during the testing period, a score of 0 was assigned. A score of 1 was assigned when one hindlimb was retracted inwards towards the abdomen for at least 50% of the period. When both hindlimbs were partially retracted inwards towards the abdomen for at least 50% of the period, the animal received a score of 2. Finally, the mice that retracted both hindlimbs completely inwards towards the abdomen for at least 50% of the observation period got a score of 3.

*Y maze*. An elevated (50 cm from the floor) Y maze shaped arena consisting of 3 arms (30 × 6 × 15 cm) was used to assess spatial memory. To evaluate *short-term working memory*, mice were placed in one arm and allowed to freely explore the Y maze for 8 min. Their movement was tracked with ANYmaze® software and the percentage spontaneous alternations (number of spontaneous alternations/(number of entries − 2) × 100) was scored manually. To evaluate *reference memory*, mice were allowed to freely explore the arena for 15 min with one of the arms closed. After a 5 min rest in a holding cage, mice were put back in the Y maze with all the arms open for 5 min. The time spent in each arm was tracked with ANYmaze® software.

*Marble burying*. Fifteen glass marbles were placed in a 3 × 5 alignment in a cage filled with a 3 cm layer of cedar wood bedding. Mice were placed in the test cage for 30 min and filmed with a camera on top of the cage. The percentage of marbles buried was analyzed using ImageJ. The number of digging bouts and duration of digging per bout was manually scored blinded for the genotype. The distance travelled and time immobile were analyzed using ANYmaze® software.

### Perfusion and tissue collection

For histology, protein and RNA extraction: mice aged 8 weeks, 12 weeks or 1 year were given an overdose of sodium pentobarbital and were transcardially perfused with saline. Gastrocnemius muscle and brain tissue were carefully isolated to determine their weight. For the brain tissue, a cut in the sagittal plane was made to obtain 2 identical hemispheres. One hemisphere was placed for 24 h in 4% paraformaldehyde (PFA) in PBS for fixation and cut the next day in 40 µm thick coronal brain slices with a vibrating microtome (HM650V, Microm). Brain slices were stored as floating sections in 0.1% (w/v) sodium azide in PBS at 4 °C. The other hemisphere was used to isolate specific brain regions (cerebellum, hippocampus, striatum, olfactory bulb and cerebral cortex) which were snap-frozen and stored at − 80 °C for protein and RNA extraction.

For histology of LEDGF KO validation, PogZ expression and expression of synaptic markers mice aged 8 weeks were given an overdose of sodium pentobarbital and transcardially perfused with saline and 4% PFA in PBS. After post-fixation for 24 h in 4% PFA in PBS, 40 µm thick sagittal brain slices were cut with a vibrating microtome. Brain slices were stored as floating sections in 0.1% (w/v) sodium azide in PBS at 4 °C.

### Immunohistochemistry

All stainings were performed on free floating sections using a wobbler. To assess the expression of both isoforms of LEDGF across the full brain sagittal sections were selected. Coronal sections were selected for the spinal cord. To evaluate the KO of LEDGF in specific type of neurons coronal or sagittal sections from the spinal cord, cerebellum or substantia nigra were selected. Antigen retrieval with 0.01 M citrate buffer of pH 6 was performed for 30 min at 80 °C. Afterwards, sections were blocked with 10% (v/v) goat or donkey serum (Dako) and 0.1% (v/v) Tergitol in PBS for 1 h and incubated with primary antibody in 10% (v/v) goat or donkey serum (Dako) and 0.1% (v/v) Tergitol in PBS overnight at room temperature. To assess the expression of both isoforms the rabbit anti-LEDGF/p75 antibody (Bethyl, A300-848A) was used in a dilution of 1/500 and the mouse anti-Psip1 antibody (Invitrogen, MA5-15,795) was used in a dilution of 1/1000. To check the KO in specific type of neurons the rabbit anti-LEDGF/p75 antibody (Bethyl, A300-848A) was used in a dilution of 1/500 and combined with one of the following antibodies: the goat anti-ChAT antibody (Millipore, AB144P; 1/100), the mouse anti-calbindin antibody (Sigma-Aldrich®, C9848; 1/1000) or the chicken anti-TH antibody (Aves Labs, TYH; 1/1000). The day after, sections were incubated with one of the following combinations of secondary antibodies: fluorochrome-conjugated goat anti-rabbit Alexa 488 (Invitrogen; 1/500) and goat anti-mouse Alexa 555 (Invitrogen; 1/500) IgG, donkey anti-rabbit Alexa 488 IgG (Invitrogen; 1/500) and donkey anti-goat Alexa 647 IgG (Invitrogen; 1/500), goat anti-mouse Alexa 488 IgG (Invitrogen; 1/500) and goat anti-rabbit Alexa 555 IgG (Invitrogen; 1/500) or goat anti-rabbit Alexa 488 IgG (Invitrogen; 1/500) and goat anti-chicken Alexa 633 IgG (Invitrogen; 1/500) for 2 h in the dark. After rinsing, sections were mounted on a glass slide and treated with TrueBlack® Lipofuscin Autofluorescence Quencher (1/20 in PBS, Biotium®) for 3 min. When the sections were completely dry they were covered with a coverslip using Mowiol (Sigma-Aldrich®).

To evaluate brain-wide neuronal densities, coronal sections of the full brain were selected every 480 µm. To assess loss of dopaminergic neurons in the substantia nigra pars compacta (SNpc), coronal sections of the SNpc were selected every 160 µm. Staining was performed using eight-section Staining Nets (Ted Pella). Antigen retrieval with 0.01 M citrate buffer at pH 6 was performed for 30 min at 80 °C. Endogenous peroxidase activity was quenched by treating the floating sections with 3% (v/v) hydrogen peroxide in PBS for 15 min. Afterwards, sections were blocked with 10% (v/v) goat serum (Dako) and 0.1% (v/v) Tergitol in PBS for 1 h and incubated with primary antibody in 10% (v/v) goat serum (Dako) and 0.1% (v/v) Tergitol in PBS overnight at room temperature. The chicken anti-NeuN antibody (Millipore, ABN91; 1/10000) or the rabbit anti-TH antibody (Millipore, AB152; 1/10000) were used as primary antibodies, respectively. The day after, sections were incubated with biotinylated goat anti-chicken IgG (Abcam, 1/1000) or biotinylated goat anti-rabbit IgG (Abcam; 1/1000) for 2 h. Sections were incubated for 1 h with the Streptavidin-HRP complex (Dako; 1/1000). The protein was visualized using 3,3-diaminobenzidine (DAB, Sigma-Aldrich®) as chromogen. Sections were mounted on glass slides and dehydrated. A coverslip was applied using DPX mounting medium (Sigma-Aldrich®).

To assess the number of MeCP2 condensates in different brain regions, coronal sections of the full brain were selected every 480 µm. Staining was performed using eight-section Staining Nets (Ted Pella). Antigen retrieval with 0.01 M citrate buffer at pH 6 was performed for 30 min at 80 °C. Afterwards, sections were blocked with 10% (v/v) goat serum (Dako) and 0.1% (v/v) Tergitol in PBS for 1 h and incubated with primary antibody in 10% (v/v) goat serum (Dako) and 0.1% (v/v) Tergitol in PBS overnight at room temperature. The rabbit anti-MeCP2 antibody (Cell Signaling, 3456S) was used in a dilution of 1/2500 and the mouse anti-Psip1 antibody (Invitrogen, MA5-15,795) was used in a dilution of 1/1000. The day after, sections were incubated with fluorochrome-conjugated goat anti-mouse Alexa 488 (Invitrogen) or goat anti-rabbit Alexa 555 (Invitrogen) IgG in a dilution of 1/500 for 2 h in the dark. After being rinsed, sections were mounted on a glass slide and treated with TrueBlack® Lipofuscin Autofluorescence Quencher (1/20 in PBS, Biotium) for 3 min. When the sections were completely dry they were covered with a coverslip using Mowiol (Sigma-Aldrich®).

To verify the expression of POGZ in the cerebellum and hippocampus, sagittal sections were used. Antigen retrieval with 0.01 M citrate buffer at pH 6 was performed for 30 min at 80 °C. Afterwards, sections were blocked with 10% (v/v) goat serum (Dako) and 0.1% (v/v) Tergitol in PBS for 1 h and incubated with primary antibody in 10% (v/v) goat serum (Dako) and 0.1% (v/v) Tergitol in PBS overnight at room temperature. The rabbit anti-POGZ antibody (Proteintech, 30,106–1-AP) was used in a dilution of 1/500 and the mouse anti-calbindin antibody (Sigma-Aldrich®) was used in a dilution of 1/1000. The day after, sections were incubated with fluorochrome-conjugated goat anti-mouse Alexa 488 (Invitrogen) or goat anti-rabbit Alexa 555 (Invitrogen) IgG in a dilution of 1/500 and DAPI (Invitrogen, 1/2000) for 2 h in the dark. After rinsing, sections were mounted on a glass slide and covered with a coverslip using Mowiol (Sigma-Aldrich®).

To determine expression of synaptic markers, sagittal sections of 40 µm were used. Sections were blocked with 10% (v/v) goat serum (Dako) and 0.1% (v/v) Tergitol in PBS for 1 h and incubated with primary antibody in 10% (v/v) goat serum (Dako) and 0.1% (v/v) Tergitol in PBS overnight at room temperature. The mouse anti-synaptophysin antibody (Millipore, MAB5258) was used in a dilution of 1/200 and the rabbit anti-PSD95 antibody (Abcam, ab18258) was used in a dilution of 1/1000. The day after, sections were incubated with fluorochrome-conjugated goat anti-mouse Alexa 488 (Invitrogen) or goat anti-rabbit Alexa 633 (Invitrogen) IgG in a dilution of 1/500 and DAPI (Invitrogen, 1/2000) for 2 h in the dark. After rinsing, sections were mounted on a glass slide and covered with a coverslip using Mowiol (Sigma-Aldrich®).

### Fluorescence image acquisition for MeCP2 condensates analysis

Brain sections of 40 µm were imaged using an AxioScan 7 microscope (Carl Zeiss AG, Germany) equipped with a Plan-Apochromat 20 × /0.8 M27 objective lens. Fluorescence imaging was performed with an Axiocam 712 Mono camera at a pixel size of 0.173 µm/pixel. The samples were illuminated using an X-Cite Xylis Lamp at 5% intensity across all channels, with an exposure time of 0.637 ms for DAPI and 100 ms for MeCP2 for both the cerebellum and hippocampus. Fluorescence excitation for DAPI was achieved with a 370–410 nm filter, and emission was captured through a 430–470 nm filter. MeCP2 Alexa Fluor 555 was detected with excitation using a 538–562 nm filter and emission captured with a 570–640 nm filter. All images were acquired using fluorescence contrast, and the light source configuration remained consistent throughout the imaging process to ensure uniform illumination. For both the cerebellum and the hippocampus, a Z-stack image was acquired and an extended depth of field (EDF) image was created. For the cerebellum the EDF was created with the variance method and no Z-stack alignment. The contrast length was set to 3, the smoothing to 5 and the reconstruction factor to 0.05. For the hippocampus the EDF was created with the variance method and the normal Z-stack alignment. The contrast length was set to 3, the smoothing to 2 and the reconstruction factor to 0.01. Both EDF as well as stitching were performed using Zen Blue version 3.7 (Carl Zeiss AG, Germany).

### Size analysis of MeCP2 condensates

MeCP2 condensate size was analyzed using a pipeline consisting of an ImageJ macro script in FIJI [29], a Groovy script in QuPath [30] and a Python notebook. The pipeline is available in the public GitHub repository (https://github.com/vib-bic-projects/202404_Speckle_Analysis). Briefly, EDFs containing MeCP2 condensates were batch processed in FIJI. The condensate signal was enhanced by subtracting the background with a rolling ball radius of 10 pixels and applying a difference of Gaussian filter with sigma values of 2 and 10. The enhanced MeCP2 signal was then batch processed in QuPath using a Groovy script with StarDist [31] for segmenting the condensates. An object classifier, trained by an expert, was additionally used to rule out any false positives, found in highly dense regions of the brain such as the hippocampus. Histograms of the area per brain region and per genotype were generated using a Python notebook.

### Analysis of NeuN and TH positive neurons

Images were acquired using an Aperio CS2 scanner (Leica) with a 20X objective. Subsequently, images were uploaded to Aiforia® Cloud for brain-wide analysis using convolutional neural networks (CNNs). Neuronal and dopaminergic cells were quantified using the Neuronal Cell Detector (NCD) and Dopaminergic Cell Detector (DCD) models as described in Barber-Janer et al. [32].

### Intensity measurements for MeCP2, PogZ, synaptophysin and PSD95

To measure MeCP2 intensity in the cerebellum and hippocampus, images acquired for the MeCP2 condensates analysis were used. The intensity of each region of interest was determined using QuPath and the installed intensity feature. To measure PogZ intensity, tilescan images were taken with a fluorescent microscope with an objective of 10X. Afterwards, the region of interest was drawn in ImageJ and the corresponding intensity was measured. To measure the synaptophysin and PSD95 intensity in the different layers of the cerebellum, images were taken with an objective of 20x. Intensity in the different cerebellar layers was measured using ImageJ.

### Western blotting

To prepare tissue lysates, samples were homogenized with a tissue homogenizer in fixed volumes (olfactory bulb: 60 µL; hippocampus: 100 µL; striatum: 100 µL; cerebellum: 100 µL and cerebral cortex: 150 µL) of RIPA buffer (10 mM Tris HCl, 140 mM NaCl, 0.1% (w/v) SDS, 0.1% (w/v) sodium deoxycholate, 1% (v/v) Tergitol, 1 mM EDTA at pH 7.4) containing PhosSTOP™ EASYpack (Roche) and complete protease inhibitors cocktail (Roche). The lysate was centrifuged for 10 min at 3000 g at 4 °C. Afterwards, the supernatant was collected and was centrifuged for 30 min at 20,000 g at 4 °C. The total protein concentration in the supernatant was determined using the BCA protein assay kit (Thermo Fischer Scientific) according to the manufacturer’s protocol. An amount of 25 µg of protein was loaded on a 4–15% Criterion™ TGX™ Precast Midi Protein Gel (Bio-Rad). After running, the gel was transferred to a nitrocellulose membrane (GE Healthcare Life Sciences) which was stained with Ponceau S solution (Sigma-Aldrich®) to check equal loading of the samples. The membrane was blocked with 5% (w/v) milk powder and PBS with 0.1% (v/v) Tergitol for 1 h and was incubated with primary antibody at 4 °C in blocking buffer overnight. The rabbit monoclonal anti-LEDGF (Abcam, 177,159; 1/400), mouse monoclonal anti-vinculin (Sigma-Aldrich®, V9131; 1/100000), rabbit polyclonal anti-GAPDH (Abcam, ab9485; 1/1000), mouse monoclonal anti-synuclein antibody (BD Biosciences, 610,786; 1/1000), rabbit monoclonal anti-synaptophysin (Abcam, ab32127; 1/1000) and the rabbit polyclonal anti-PSD95 (Abcam, ab18258; 1/1000) were used. The day after, the membrane was incubated for 2 h at room temperature with horseradish peroxidase-conjugated secondary antibodies anti-rabbit (Dako, 1/20000) and anti-mouse (Dako, 1/20000) IgG. Chemiluminescent detection was performed with the Clarity Western Blot ECL Substrate (Bio-Rad) or Clarity Max Western ECL Substrate (Bio-Rad) using an Amersham ImageQuant 800 biomolecular imager (GE Healthcare Life Sciences).

### RNA isolation, library preparation and sequencing

Total RNA was extracted from mouse brain tissue using Aurum™ Total RNA Mini Kit (Bio-Rad) followed by column purification according to the manufacturer’s protocol. RNA purity and concentration was assessed using Implen NanoPhotometer NP80 Touch (Westburg). RNA integrity was assessed using the Bioanalyzer 2100 system (Agilent Technologies) and samples with RNA Integrity number (RIN) > 7 were retained. RNA sequencing libraries were prepared using Novogene NGS Stranded RNA Library Prep Set (PT044). Library quality was checked with Qubit fluorometer (Thermo Fisher Scientific) and real-time PCR for quantification and Bioanalyzer 2100 system (Agilent Technologies) for size distribution detection. After library quality control, different libraries were pooled based on the effective concentration and targeted data amount, then subjected to Illumina NovaSeq X Plus strand-specific sequencing with a sequencing depth of 30 million reads.

### RNA sequencing data pre-processing, quality control and alignment

Raw sequencing reads were obtained in FASTQ format with a minimum of 9 million reads per sample. Raw files were processed using fastp for quality filtering and adapter trimming. Read with adapter sequences, poly-N stretches, and low quality (thresholds) were removed. Quality of the filtered reads was assessed via Q20, Q30 and GC content. All downstream analysis were performed on these high-quality clean reads.

Paired-end reads were aligned to the mouse reference genome (mm39) using the splice-aware alignment tool HISAT2 (version 2.0.5) default parameters. FeatureCounts v1.5.0-p3 was used to count the reads numbers mapped to each gene. The resulting count matrix was imported in R (v4.3.2) for downstream analysis.

### Differential gene expression analysis

Samples from the either brain region (cerebellum or hippocampus) were selected based on metadata. Genes with fewer than 10 reads across all samples were filtered out. Differential gene expression was performed using DESeq2 (v1.40.2) comparing conditional knockout (cKO) to wild-type (WT) mice. Variance stabilizing transformation (VST) was applied for visualization. DEGs were defined by an adjusted p-value < 0.001 and absolute log₂ fold change > 0.75. Ensembl gene IDs were converted to gene symbols using the org.Mm.eg.db (v3.17.0).

### Functional and regulatory network enrichment

DEGs for functional analysis were defined as genes with |log₂ fold change|> 0.75 and adjusted p-value < 0.01, obtained from DESeq2 (v1.40.2). GO overrepresentation analysis was performed using enrichGO, for all ontologies: Biological Process (BP), Molecular Function (MF), and Cellular Component (CC) ontologies. Enriched GO terms were considered significant at adjusted p-value < 0.05 (Benjamini–Hochberg correction). Cnetplot was used highlight gene uniqueness and association among enriched categories. To complement overrepresentation analysis, Gene Set Enrichment Analysis (GSEA) was performed using via clusterProfiler. In brief, genes were pre-ranked by log₂ fold change, and gene sets with sizes between 10 and 500 genes were tested. Significantly enriched gene sets were considered at adjusted p-value < 0.05. Transcription factor and histone modification enrichment was evaluated for DEG genes via enrichR (v3.1) querying *ChEA_2022* database, and *ENCODE_Histone_Modifications_2015,* respectively.

### Reverse transcription quantitative polymerase chain reaction

A total amount of 5 μg RNA was used to perform reverse transcription with the High-capacity cDNA Archive Kit (Applied Biosystems). SsoAdvanced Universal SYBR Green Supermix (Bio-Rad) was used to perform reverse transcription quantitative polymerase chain reaction (RT-qPCR) with the CFX Opus 96 Real-Time PCR System (Bio-Rad) as detection system. Primers for GAPDH were used to normalize the mRNA levels. *Snca*: forward 5’- GGGGTACCCACAGGAAGGAA-3’ reverse 5’-CATAAGCCTCACTGCCAGGAT-3’. *Gapdh*: forward 5’-TGTGTCCGTCGTGGATCTGA-3’ reverse 5’-CCTGCTTCACCACCTTCTTGA-3’.

### Statistical analysis

All statistical analysis was performed using GraphPad Prism 10. Details of the specific statistical tests, p-values and number of biological replicates are provided in the figure legend. All results are shown as mean ± SEM and individual values.

## Results

### Brain-region dependent expression of LEDGF isoforms

**Fig. 1:**
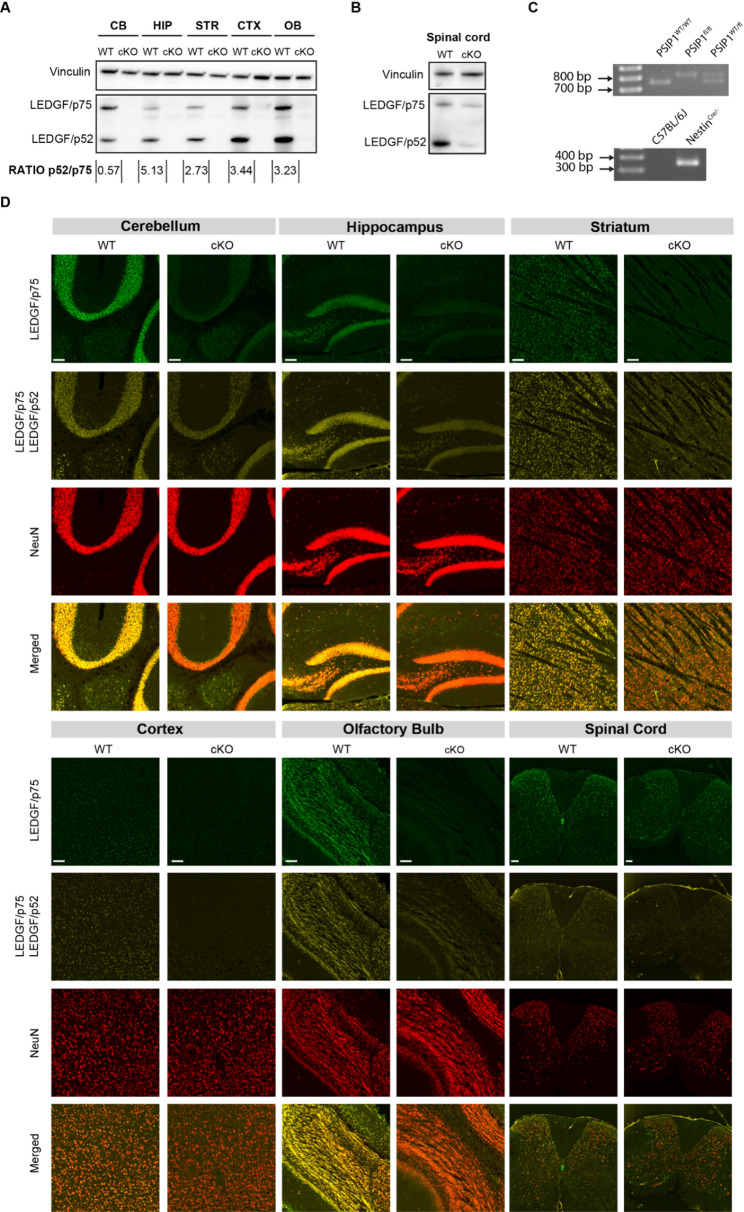
Validation of *Psip1 c*KO in the central nervous system. **A** Western Blot for LEDGF and vinculin expression in different brain regions from 12 week old animals. n = 4 for each brain region to calculate the ratio of the isoforms. **B** Western Blot for LEDGF and vinculin expression in spinal cord from 8 week old animals. **C** Validation of genotype on genomic DNA. Top panel: PCR for *Psip1*, resulting in a band of 745 bp for *Psip1*^WT/WT^ and a band of 802 bp for *Psip1*^fl/fl^ mice. Bottom panel: PCR for Cre, resulting in a band of 350 bp for Nestin^Cre/−^ mice. **D** Staining of different brain regions and spinal cord for LEDGF/p75 specifically or LEDGF/p75 & LEDGF/p52 in WT and cKO animals at an age of 8 weeks. NeuN was used as a neuronal marker. Merged images highlight the co-localization of LEDGF and NeuN. CB = cerebellum; HIP = hippocampus; STR = striatum; CTX = cortex and OB = olfactory bulb. Scale bar is 100 µm. Images are taken with a fluorescence microscope with the 5X objective.

LEDGF expression was determined by Western Blotting of protein extracts from the cerebellum, hippocampus, striatum, cerebral cortex and olfactory bulb from WT animals. Using an antibody that detects both isoforms of LEDGF (p75 and p52), we were able to compare the ratio of the isoforms in different brain regions (Fig. 1A). In the cerebellum a twofold higher level of LEDGF/p75 was detected, whereas the hippocampus displayed fivefold more LEDGF/p52 than LEDGF/p75. The highest expression of LEDGF/p75 was observed in the cerebellum, while LEDGF/p52 showed the highest expression in the cortex and olfactory bulb (Fig. S1A-B). When examining the total LEDGF expression across various brain regions in WT animals, the cerebellum, olfactory bulb, and cortex exhibited the highest levels (Fig. S1C). Immunohistochemistry with 2 distinct antibodies that allowed to detect either only LEDGF/p75 or both isoforms, revealed that both isoforms are expressed across the brain, with the highest levels in the cerebellum, hippocampus and olfactory bulb (Fig. 1D). The signals from both LEDGF antibodies coincided with NeuN staining, indicating that LEDGF expression is enriched in neurons (Fig. 1D).

### Generation and validation of CNS-specific *Psip1* cKO mice

*Psip1*^fl/fl^ mice carrying loxP sites around exon 3 of the *Psip1* gene were previously generated [11]. Homozygous *Psip1*^fl/fl^ mice were crossbred with heterozygous Nestin^Cre/−^ mice to obtain a KO of *Psip1* in the central nervous system (CNS) (see Methods sections), hereafter referred as *Psip1* cKO mice. After weaning, each mouse was genotyped for *Psip1* and *Nestin*^*Cre*^ genes prior to proceeding with further experimentation (Fig. 1C). Western Blotting revealed the KO of both LEDGF isoforms in different brain regions (Fig. 1A). Moreover, a reduction in LEDGF signal was observed in the spinal cord of cKO mice (Fig. 1B). KO of both isoforms in neurons was also investigated by immunohistochemistry (Fig. 1D). In the spinal cord, a reduction of LEDGF was confirmed in the dorsal horn by staining. However, both LEDGF isoforms were still present in motor neurons of cKO mice. To validate the CNS-specificity of LEDGF depletion, expression of LEDGF in the liver and kidney was verified with Western Blotting. No reduction in LEDGF expression was detected in either of these organs (data not shown).

### *Psip1* cKO mice show hypolocomotion and impairment in motor coordination and balance

In the group monitored for 1 year, we determined the body weight every 3 months. No body weight differences between WT and cKO mice were observed (Fig. S2A). In 1-year old animals, both the brain and gastrocnemius muscle were isolated and weighed to assess differences in tissue mass between WT and cKO animals. No weight difference between both groups in either brain or gastrocnemius muscle were detected (Fig. S2B-C).

**Fig. 2:**
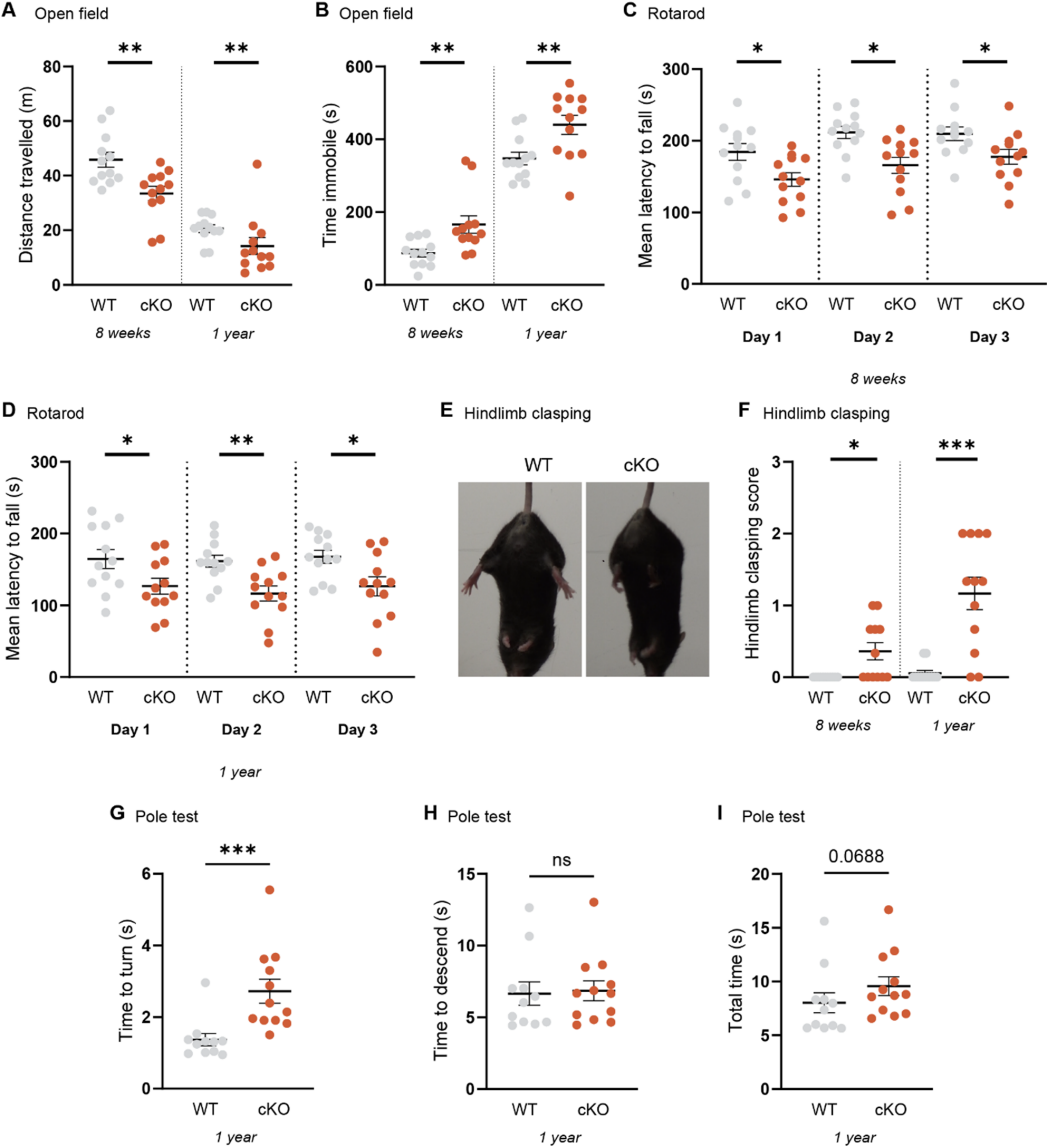
*Psip1* cKO mice show hypoactivity and impaired motor coordination and balance starting at an age of 8 weeks. **A** Distance travelled and **B** time spent immobile in the open field test at 8 weeks and 1 year of age. **C** Mean latency to fall off the rod on 3 consecutive days at 8 weeks. **D** Mean latency to fall off the rod on 3 consecutive days at 1 year of age. **E** Example of hindlimb clasping present in a cKO mouse of 1 year of age. **F** Hindlimb clasping score for WT and cKO mice at 8 weeks and 1 year of age. **G** Time to turn, **H** time to descend and **I** total time needed in the pole test at an age of 1 year. Mann–Whitney test (**A**,**B**,**F**-**I**) or unpaired t-test (**C**,**D**) with n = 12 for each genotype, except for pole test with n = 11 for WT and n = 12 for cKO. ns not significant, *p < 0.05, **p < 0.01, ***p < 0.001. Data are presented as mean ± SEM and individual values.

After validation of brain-wide depletion of neuronal LEDGF both by Western Blotting (Fig. 1A) and immunohistochemistry (Fig. 1D), we performed a series of behavioral tests. We first assessed general motor performance by performing the open field test at an age of 8 weeks and 1 year. The *Psip1* cKO mice were characterized by a significant decrease in the distance travelled and an increase in time spent immobile, pointing towards hypolocomotion (Fig. 2A and B, Table 1). Given the observed motor phenotype, we decided to perform the rotarod test to evaluate motor coordination and balance. Already at an age of 8 weeks, cKO mice showed a significant decrease in the time spent on the rod on the first day of the test, which remained consistent on the second and third day of the test (Fig. 2C, Table 1). At an age of 1 year, cKO mice still spent less time on the rod on all days of the test (Fig. 2D, Table 1). Given the prevalence of hindlimb clasping in RTT mouse models, we also examined clasping behavior in our mouse model. At 8 weeks of age, there was a slight but statistically significant increase in the hindlimb clasping score (Fig. 2F, Table 1). Hindlimb clasping was more pronounced and severe at an age of 1 year pointing towards aggravating clasping phenotype with age (Fig. 2E and F, Table 1). To gain more insight in motor coordination and balance, the pole test was conducted at an age of 1 year. cKO mice required significantly more time to turn when placed on the pole (Fig. 2G, Table 1). Although there was no significant difference in the time to descend the pole, there was a trend (*p* = 0.088) towards a longer total time needed for the test (Fig. 2H and I, Table 1). The results of the pole test are consistent with the deficits in motor coordination observed on the rotarod in cKO mice.

**Table 1:** Overview results behavioral phenotyping *Psip1* cKO versus WT mice

	8 weeks		1 year	
	*WT*	*cKO*	*WT*	*cKO*
Motor related				
*Open field*				
Distance travelled (m)	45.8 ± 2.73	33.47 ± 2.63^**^	20.6 ± 1.42	14.3 ± 3.1^**^
Time immobile (s)	87.4 ± 10.62	165.7 ± 24.0^**^	347.4 ± 17.5	439.6 ± 26.1^**^
*Rotarod*				
Latency to fall (s)				
Day 1	184.4 ± 11.68	145.8 ± 9.6^*^	164.4 ± 13.3	126.7 ± 11.0^*^
Day 2	211.7 ± 8.5	165.7 ± 11.3^*^	161.3 ± 8.3	116.5 ± 10.4^**^
Day 3	209.6 ± 9.3	177.5 ± 10.3^*^	167.4 ± 9.1	126.4 ± 13.3^*^
*Hindlimb clasping*				
Clasping score	0.0 ± 0.0	0.4 ± 0.1^*^	0.1 ± 0.03	1.2 ± 0.2^***^
*Pole test*				
Time to turn (s)	NT	NT	1.4 ± 0.2	2.7 ± 0.3^***^
Time to descend (s)	NT	NT	6.7 ± 0.8	6.8 ± 0.7
Total time (s)	NT	NT	8.0 ± 0.9	9.6 ± 0.9
Non-motor related				
*Open field*				
Time in center (s)	43.8 ± 5.4	27.3 ± 3.8^*^	33.9 ± 3.8	12.0 ± 4.3^**^
Entries in center	28.9 ± 2.0	16.3 ± 1.6^****^	17.6 ± 2.2	5.0 ± 1.5^****^
*Elevated Plus Maze*				
Time in open arm (%)	19.7 ± 2.8	10.3 ± 3.0^*^	10.3 ± 1.6	8.6 ± 2.5
*Marble burying*				
Marbles buried	NT	NT	9.3 ± 1.0	3.8 ± 1.2^**^
Digging bouts	NT	NT	78.1 ± 7.4	48.4 ± 6.7^**^
Total time digging (s)	NT	NT	199.5 ± 28.0	124.3 ± 18.2^*^
*Y maze*				
SAR (%)	57.9 ± 2.7	62.8 ± 3.9	61.5 ± 1.8	51.0 ± 3.9^*^
Time in novel arm (%)	49.0 ± 4.6^##^	42.3 ± 2.6^##^	45.5 ± 4.7^#^	39.5 ± 4.4

Data presented as mean ± SEM; NT: not tested; significantly different from WT: ^*^p < 0.05, ^**^p < 0.01, ^***^p < 0.001, ^****^p < 0.0001; significantly different from hypothetical value of 33.33%: ^#^*p* < 0.05, ^##^*p* < 0.01

### *Psip1* cKO mice display an anxiety phenotype

**Fig. 3:**
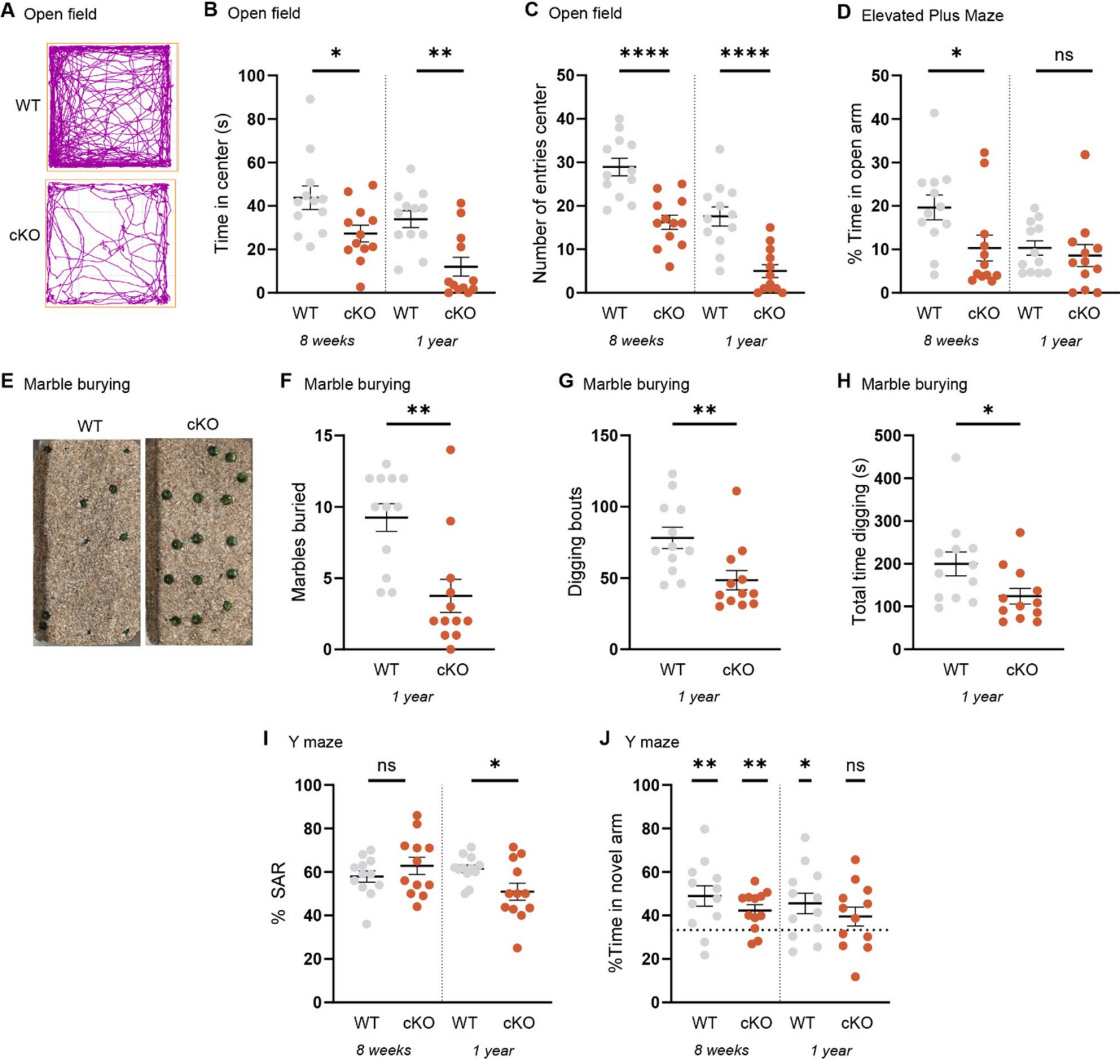
Anxiety-related behavior and memory deficits in *Psip1* cKO mice. **A** Track plot of a WT and cKO mouse at an age of 8 weeks in the open field test. **B** Time spent in the center in the open field test. **C** Number of entries in the center in the open field test. **D** Percentage of time spent in the open arms in the elevated plus maze. **E**–**F** Number of marbles buried in the marble burying test. **G** Number of digging bouts and **H** total time digging in the marble burying test. **I** Spontaneous alternation (SAR) percentage in the Y maze test. **J** Percentage time spent in the novel arm in the delayed Y maze test. Mann–Whitney test (**B**, **D**, **F**–**I**) or unpaired t-test (**C**) versus WT at each indicated time point with n = 12 for each genotype. One sample t-test (**J**) against hypothetical value of 33.33% (dashed line, equal chance of arm entry in the novel compared to familiar arms) with n = 12 for each genotype. ns not significant, * p < 0.05, ** p < 0.01, **** p < 0.0001. Data are presented as mean ± SEM and individual values.

After examining the motor phenotype, we implemented a series of other tests to provide a more thorough evaluation of the behavioral phenotype. First, we evaluated anxiety-related behavior in the open field test by tracking the time spent in the center and the number of entries in the center. Already at an age of 8 weeks, cKO mice showed a significant reduction in the time spent in the center and the number of entries in the center compared to their WT controls (Fig. 3A–C, Table 1). The same result was obtained in the group of 1 year (Fig. 3A–C, Table 1). Next, we performed the elevated plus maze test which is another widely used test to assess anxiety-related behavior [33]. At an age of 8 weeks, cKO mice spent less time in the open arms than WT controls, but this difference was not observed anymore at 1 year of age (Fig. 3D, Table 1). The marble burying test is often used in literature to determine anxiety-related or repetitive behavior [34]. However, in the field of RTT, the marble burying test is also used to assess exploratory behavior [35]. Animals were tested at an age of 1 year revealing a reduction in the number of marbles buried in the cKO mice, pointing towards a reduction in exploratory behavior (Fig. 3E and F, Table 1). In addition cKO mice showed less digging bouts, resulting in a significant lower total time digging (Fig. 3G and H, Table 1) without a significant reduction in total locomotion (data not shown).

### *Psip1* cKO mice show spatial memory deficits at an age of 1 year

As part of the assessment of the behavioral phenotype, two variants of the Y maze test were performed to evaluate spatial memory. In the first variant, mice were placed in the Y maze and allowed to freely explore the maze for a fixed period of time. The spontaneous alternation score was calculated, revealing a decrease in spontaneous alternations at an age of 1 year, but not at 8 weeks in cKO mice (Fig. 3I, Table 1). These findings indicate a deficit in the short-term working memory occurring at an older age in cKO mice. In the second variant, mice were placed in the Y maze with one of the three arms closed. After the training phase, mice were given a period of rest and were replaced in the Y maze where all three arms were accessible. The time spent in the novel and familiar arm was recorded. The cKO mice were still able to distinguish the novel arm from the familiar arm at an age of 8 weeks (Fig. 3J, Table 1). However, this was not the case at an age of 1 year, indicating impairments in spatial reference memory in cKO mice at older age (Fig. 3J, Table 1).

### In *Psip1* cKO mice LEDGF is depleted in dopaminergic neurons, but not in motor neurons of the spinal cord nor in Purkinje cells in the cerebellum

The pronounced motor phenotype made us investigate potential neurodegeneration across different brain regions in the *Psip1* cKO mice at 12 weeks and 1 year of age. We employed the Neuronal Cell Detector (NCD) model developed to perform brain-wide neuronal cell quantification using the neuronal marker NeuN [32, 36]. Analysis of the NeuN positive areas in multiple brain regions showed no significant neuronal loss at 12 weeks or at 1 year in the *Psip1* cKO mice compared to WT mice (Fig. S3A-H).

**Fig. 4:**
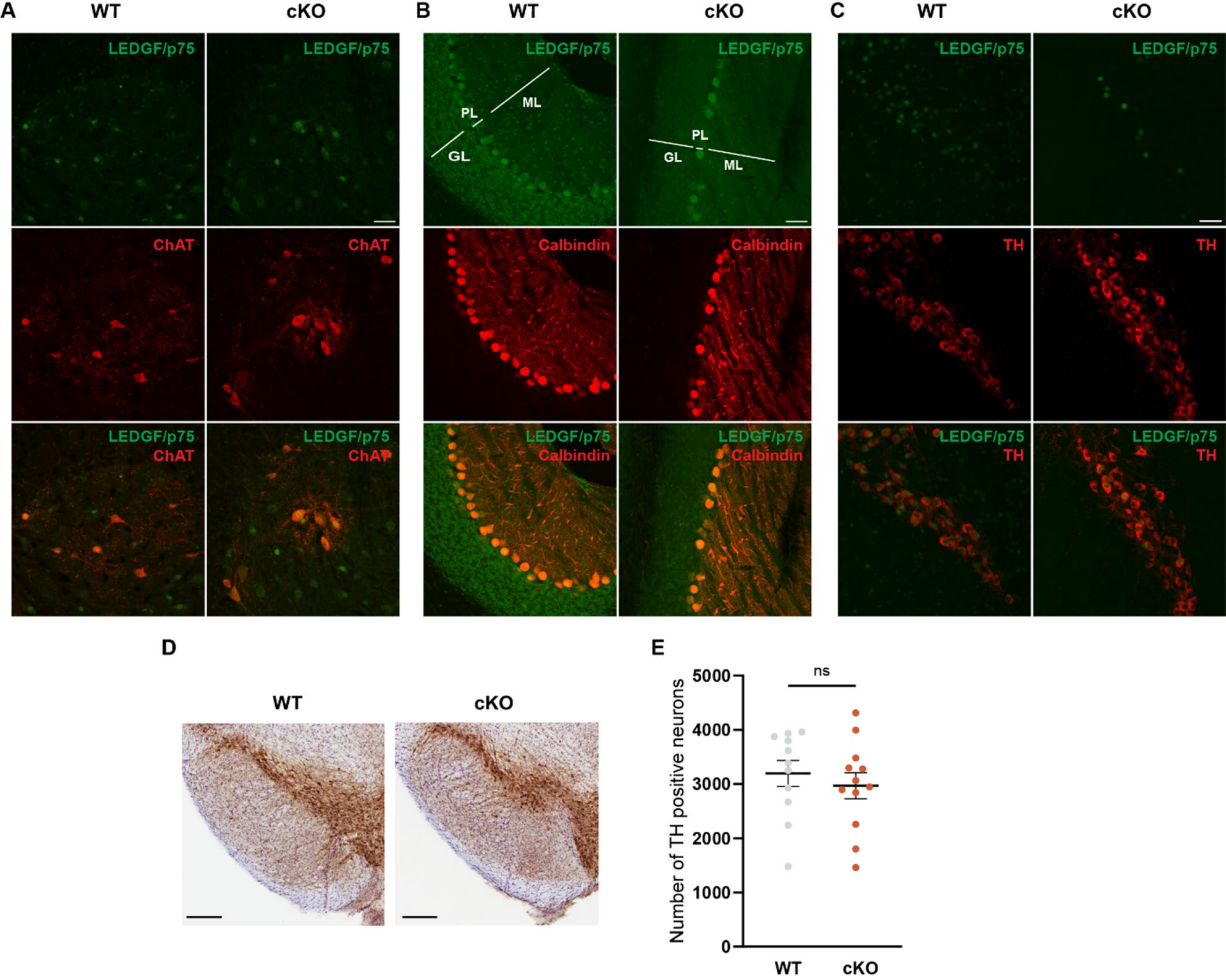
Expression of LEDGF/p75 in specific neuronal subtypes. **A** Co-staining for choline acetyltransferase (ChAT) and LEDGF/p75 in motor neurons of the spinal cord of WT and cKO mice. **B** Co-staining for calbindin and LEDGF/p75 in the Purkinje cell layer of the cerebellum of WT and cKO mice. ML = granular layer; PL = Purkinje cell layer; GL = granular layer **C** Co-staining for tyrosine hydroxylase (TH) and LEDGF/p75 in dopaminergic neurons in the substantia nigra pars compacta in WT and cKO mice. **D** Example of TH staining in the substantia nigra of WT and cKO mice of 1 year of age. **E** Number of TH positive neurons in the right hemisphere of WT and cKO mice at 1 year of age. Unpaired t-test with n = 8 for WT/n = 9 for cKO. Data are presented as mean ± SEM and individual values. Scale bar is 50 µm. Images taken with confocal microscope with 20X objective.

In view of the apparent motor phenotype, we further focused on 3 neuronal subtypes critical in the regulation of motor function: motor neurons in the spinal cord, Purkinje cells in the cerebellum and dopaminergic neurons in the substantia nigra pars compacta (SNpc), respectively [37–39]. To deduce which cells express Cre from the nestin promoter, we verified LEDGF expression in these specific neuronal populations. To detect motor neurons in the spinal cord or Purkinje cells in the cerebellum, an antibody against choline acetyltransferase (ChAT) or calbindin was used respectively. No depletion of LEDGF in the motor neurons of the spinal cord (Fig. 4A) nor in Purkinje cells in the cerebellum was detected (Fig. 4B). By using an antibody against tyrosine hydroxylase (TH), we were able to detect dopaminergic neurons in the SNpc. Staining for LEDGF revealed a reduction in the dopaminergic neurons of *Psip1* cKO mice (Fig. 4C), which made us investigate the effect on the integrity of this cell type. We determined the number of TH positive neurons in animals at 1 year of age with the Dopaminergic Cell Detector (DCD) [32], but no difference in the number of TH positive neurons between WT and cKO mice was detected at an age of 1 year (Fig. 4D and E). Furthermore, we observed no neuronal loss in the primary motor cortex at 12 weeks or 1 year of age (Fig. S3B & F).

### LEDGF depletion increases the number of MeCP2 condensates in the cerebellum

**Fig. 5:**
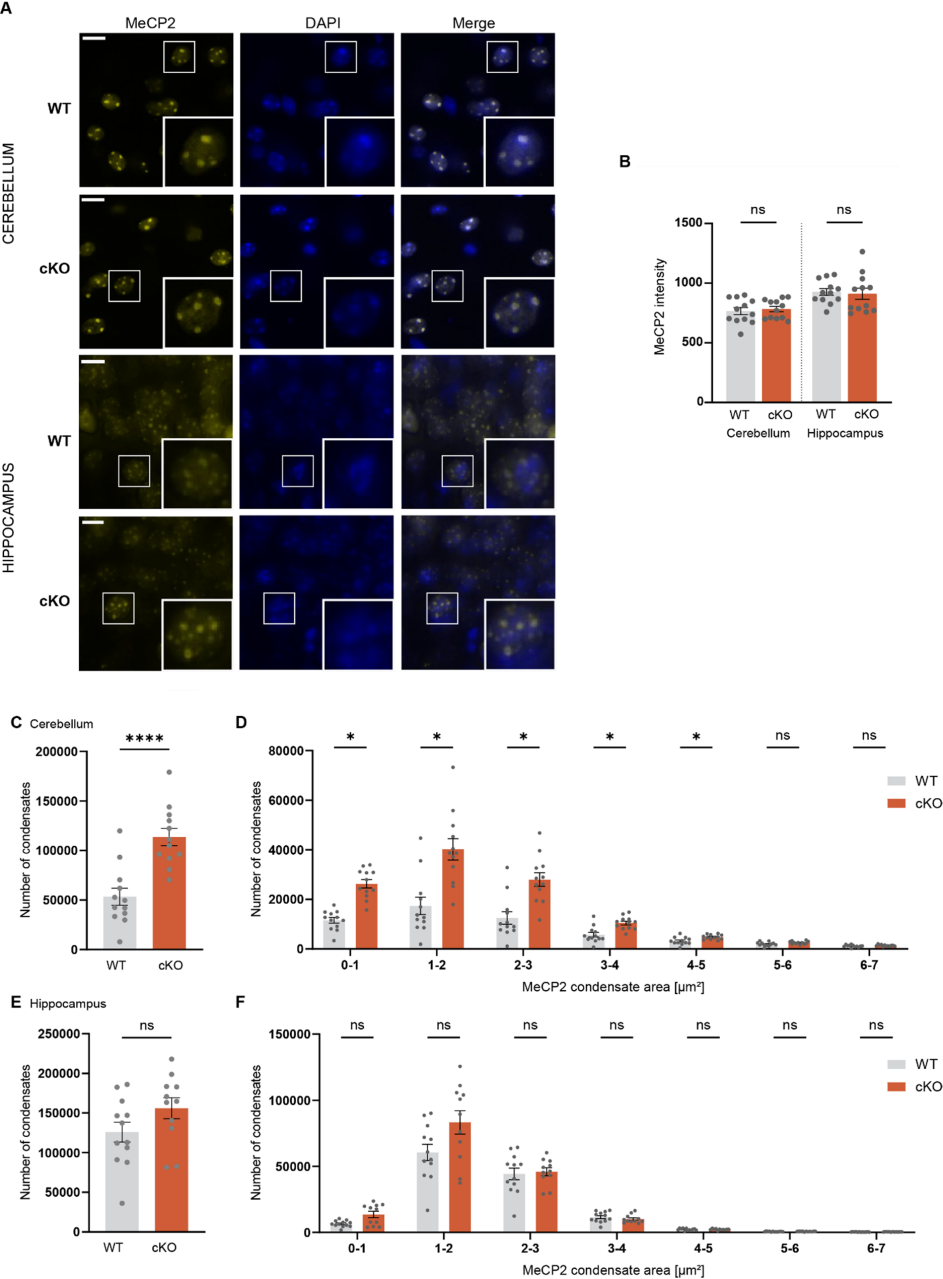
LEDGF depletion increases the number of MeCP2 condensates in mouse cerebellum. **A** MeCP2 expression and condensates in cerebellum or hippocampus in WT and cKO mice at 12 weeks of age. Scale bar is 10 µm. **B** Quantification of MeCP2 expression measured as mean intensity in the cerebellum and hippocampus. Unpaired t-test with n = 12 for each genotype. **C** Total number of condensates detected in the cerebellum of WT and cKO mice. **D** Number of condensates across different condensate area bins in both WT and cKO mice in the cerebellum. **E** Total number of condensates detected in the hippocampus of WT and cKO mice. **F** Number of condensates across different area bins in both WT and cKO mice in the hippocampus. Unpaired t-test with Holm-Sidaks correction for multiple comparisons testing with n = 12 for each genotype (except n = 11 for cKO mice in E, F). Data are presented as mean ± SEM and individual values.

Since both p52 and p75 isoforms of LEDGF are known to directly interact with MeCP2 [7–9], and since mutations in *MECP2* cause RTT associated with motor and Parkinsonian-like symptoms as well as hindlimb clasping [15], we studied the impact of LEDGF depletion on MeCP2 in the cKO mice. No change in MeCP2 intensity in the cerebellum nor in the hippocampus was seen by immunohistochemistry (Fig. 5A and B). MeCP2 is present in mouse brain cells in liquid-like heterochromatin condensates, visible as nuclear condensates across the mouse brain [40]. To analyze MeCP2 condensates in brain tissue, we created an automated analysis method to detect and measure the area of each individual speckle within a region of interest. Considering the different ratios of p52 and p75 isoforms in the cerebellum and the hippocampus (Fig. 1C), we looked at the distribution of the MeCP2 condensate number and size in these regions of WT and cKO mice (Fig. 5A). In the cerebellum, an increase in the number of MeCP2 condensates was observed in cKO mice with an increase in the number of condensates between 1 and 5 µm^2^ (Fig. 5C and D). Moreover, in the hippocampus there was also a non-significant trend towards more and smaller MeCP2 condensates in cKO mice (Fig. 5E and F).

### LEDGF depletion has no effect on nuclear localization of PogZ

PogZ is a p75-specific binding partner of LEDGF present in the nucleus of brain cells and tethered to chromatin by LEDGF/p75 [5, 17]. Given that mutations in the *POGZ* gene are associated with the White-Sutton syndrome and that *PogZ*-deficient mice display motor deficits in the rotarod test, we explored whether LEDGF modulates the expression and nuclear localization of PogZ. First, the impact of a *Psip1* KO on the expression level of PogZ in the cerebellum and the hippocampus was assessed. No significant differences in PogZ expression were observed between WT and cKO mice in either of the brain regions (Fig. S4C). Second, because some mutations in *PogZ* lead to a truncated protein that lacks the LEDGF/p75-binding domain and consequently results in a cytoplasmic distribution, we hypothesized that a *Psip1* KO might also result in more cytoplasmic expression of PogZ. Therefore, we compared the nuclear localization of PogZ in the hippocampus and the cerebellum of WT and cKO mice. In the hippocampus the nuclear localization of PogZ was evidenced in both WT and cKO mice (Fig. S4A). An antibody against calbindin, that labels Purkinje cells, was used to be able to distinguish the different layers in the cerebellum. Similarly to the hippocampus, staining revealed no difference in the nuclear localization of PogZ between WT and cKO mice in either of the layers of the cerebellum (Fig. S4B).

### LEDGF as a key regulator of chromatin-modifying complexes and synaptic gene expression in the cerebellum

**Fig. 6:**
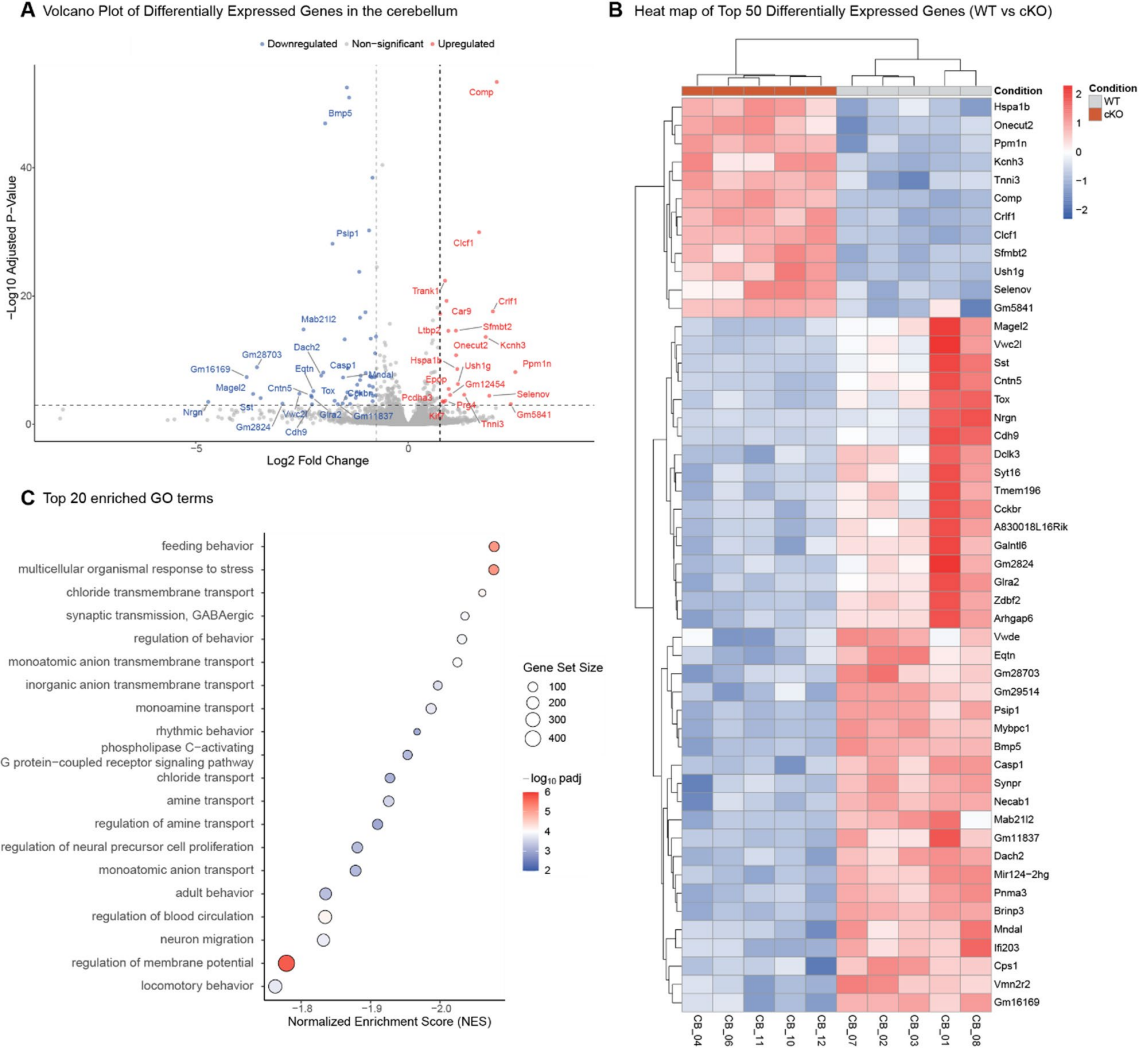
LEDGF as a key regulator of chromatin-modifying complexes controlling synaptic gene expression. **A** Volcano plot showing differentially expressed genes in the cerebellum with │log2 fold change│ > 0.75 and adjusted p-value < 0.001. **B** Heat map of the top 50 differentially expressed genes in the cerebellum. Plotted as WT vs cKO mice. The color corresponds to the scaled expression levels (blue–negative, red–positive). with │log2 fold change│ > 0.75 and adjusted *p* value < 0.001. **C** Dot plot of the top 20 enriched GO terms in GSEA analysis, ranked by normalized enrichment score (NES) with │log2 fold change│ > 0.75 and adjusted *p* value < 0.01.

Finally, to determine the pathways affected by a LEDGF depletion in mouse brain, we performed bulk RNA-sequencing on extracts of the cerebellum or the hippocampus from WT and cKO mice. We selected these brain regions based on the behavioral phenotype and the differential splicing of LEDGF in both regions. Transcriptomic analysis revealed a modest effect of LEDGF depletion in the hippocampus with only 15 downregulated and 7 upregulated genes (Fig. S5). Transcriptomic profiling of the cerebellum revealed downregulation of 53 genes and upregulation of 21 genes, confirming the role of LEDGF as a transcriptional co-activator (Fig. 8A). Heatmap visualization of the top 50 differentially expressed genes (DEGs) in the cerebellum emphasized this asymmetry with a greater proportion of downregulated genes in the *Psip1* cKO mice compared to upregulated genes. Additionally, hierarchical clustering of the top 50 differentially expressed genes resulted in clear separation between WT and cKO samples, indicating a signature associated with LEDGF loss (Fig. 6B). As expected, *Psip1* is present as most significantly downregulated gene in the cKO mice, further validating the bulk analysis in a brain region where not all cells are depleted for LEDGF (Fig. 6B). Gene Ontology (GO) analysis revealed that LEDGF depletion in the cerebellum primarily affects pathways involved in synaptic transmission, assembly and synaptic membrane components (Fig. S6A-B). Regarding molecular function, DEGs were significantly enriched in pathways associated with ion channel activity in the cerebellum of *Psip1* cKO mice (Fig. S6C). Gene Set Enrichment Analysis (GSEA) revealed significant enrichment of gene sets related to synaptic transmission, behavior, positive regulation of DNA binding and response to stress (Fig. 6C & Fig. S7).

**Fig. 7:**
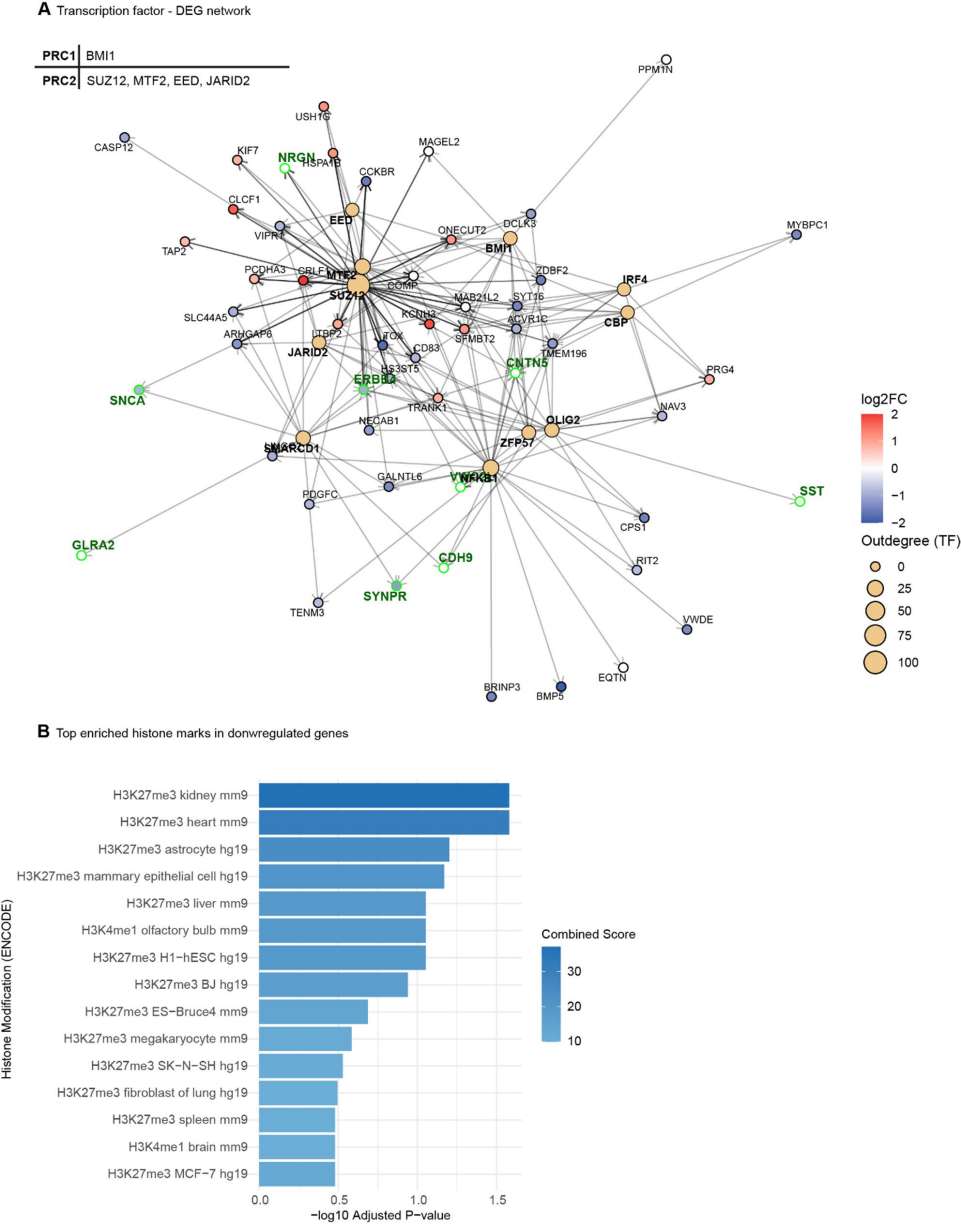
Polycomb group proteins are the main regulators of differentially expressed genes in the cerebellum. **A** Transcription factor network analysis. Transcription factors presented in bold and with yellow dots. Red dots represent upregulated genes, blue dots represent downregulated genes. Synaptic genes presented with green dots. Transcription factors that are part of the polycomb repressive complex 1 (PRC1) or polycomb repressive complex 1 (PRC2) are listed in the table. **B** Enrichment of histone marks in downregulated genes. Adjusted *p* value < 0.001.

To understand the potential upstream regulators of these transcriptional changes, we performed transcription factor (TF) enrichment analysis against the ChEA database [41]. Among the top 10 regulators of DEGs in the cerebellum of *Psip1* cKO mice, we identified one component of the Polycomb Repressive Complex 1 (PRC1): polycomb group RING finger protein 4 (BMI1) (Fig. 7A). Notably, four of the top 10 regulators are part of the Polycomb Repressive Complex 2 (PRC2), namely Metal Response Element Binding Transcription Factor 2 (MTF2), Jumonji and AT-Rich Interaction Domain Containing 2 (JARID2), Suppressor Of Zeste 12 Protein Homolog (Suz12), and embryonic ectoderm development (EED) (Fig. 7A). Polycomb group (PcG) proteins are mediating gene silencing as opposed to Trithorax group (TrxG) proteins [42]. Another important regulator of the DEGs in the cerebellum is SWI/SNF Related BAF Chromatin Remodeling Complex Subunit D1 (SMARCD1), one class of TrxG proteins (Fig. 7A). Moreover, when looking at down- and upregulated genes separately, Suz12 is the key transcription factor that regulates both down- and upregulated genes in the cerebellum of *Psip1* cKO mice (Fig. S8A-B). Notably, synaptic genes are predominantly downregulated in the cerebellum of *Psip1* cKO mice, represented as green dots in the transcription factor network analysis (Fig. S8B). One of these synaptic genes is *Snca* encoding the alpha-synuclein protein*,* for which downregulation was validated on mRNA and protein level (Fig. S9A-B). Finally, when looking at the top enriched histone marks of the downregulated genes, enrichment of the H3K27me3 mark, a typical hallmark of PRC2 activity is evidenced (Fig. 7B) [43]. The expression levels of the presynaptic marker synaptophysin and the postsynaptic marker PSD95 in the cerebellum were not significantly different between WT and cKO animals (Fig. S10), arguing against a general loss of synaptic markers, and suggesting more specific changes in genes related to synaptic transmission.

## Discussion

### The ratios of both LEDGF isoforms depend on the brain region

Given that a full body KO of *Psip1* resulted in high perinatal lethality [26], we created a conditional *Psip1* KO in the central nervous system. With WT expression of LEDGF during development until embryonic day 10.5, no perinatal lethality or abnormal skeletal transformations were observed in cKO mice [44]. Since the Nestin^Cre^ driver line has a reduced body weight due to the insertion of the human growth hormone minigene downstream of Cre recombinase, Nestin^Cre/−^ mice, referred to as WT mice, were used as a control [45].

Unexpectedly, we discovered that the ratio of the LEDGF isoforms, p75 and p52 is dependent on the brain region. This finding has functional implications. Whereas MeCP2 binds both isoforms, MLL1, PogZ and CDA7L bind specifically to the C-terminus of LEDGF/p75 [5, 7–9, 17, 19, 21]. Ge et al. previously reported that LEDGF/p52 is highly expressed in testis, thymus and brain, whereas LEDGF/p75 displays high expression in the thymus, followed by the ovary and skeletal muscle [46]. However, the ratio between both isoforms has not been verified nor specifically analyzed in distinct brain regions. Our data demonstrate alternative splicing of LEDGF in mouse brain. Alternative splicing is known to occur in the brain, contributing to neurogenesis and brain development [47]. The differential splicing may attribute a distinct p75-specific functionality to LEDGF in the cerebellum in contrast to other brain regions with predominant p52 expression. Of note LEDGF has by itself been implicated in splicing [48, 49]. A second consequence of this finding relates to the susceptibility to lentiviral vector transduction for the generation of animal models or gene therapy. LEDGF/p75 tethers and targets lentiviral preintegration complexes to active genes [50, 51]. A low level of p75 in some brain regions may result in low transduction efficiency as LEDGF/p75-specific knockdown is known to reduce lentiviral vector transduction in cell culture [52].

The cellular specificity in our study is in line with the findings from Chylack et al. who discovered strong LEDGF/p75 expression in neurons. Using NeuN as a marker, we evidenced a neuronal pattern of LEDGF/p75 and LEDGF/p52 expression in the brain of 12-week-old animals (Fig. 1D). As reported before by McGovern et al. using the Nestin Cre-loxP system, no depletion of LEDGF in the motor neurons of the spinal cord was found (Fig. 1D) [53]. However, we detected a reduction in LEDGF signal in the dorsal horn of the spinal cord, an area that is involved in the processing of somatosensory information [54].

### *Psip1* cKO mice show motor function impairments

Our aim was to screen for motor and non-motor phenotypes through an extensive behavioral phenotyping. This phenotyping revealed a pronounced motor phenotype especially in the open field and rotarod tests (Fig. 2A-D, Table 1). In agreement with the few surviving full body *Psip1* KO mice of Sutherland et al., we also observe reduced locomotor activity in the open field test and hindlimb clasping which is becoming more pronounced with age [26] (Fig. 2A-B; Fig. 2E-F, Table 1). Of note, hindlimb clasping is also present in RTT mouse models. In the pole test, we mainly noticed problems in turning downwards to descend the pole (Fig. 2G-I, Table 1). The pole test is a commonly used test in the field of Parkinson’s disease to assess sensorimotor impairments [55]. The results of the open field test suggest increased anxiety in the *Psip1* cKO mice (Fig. 3A-C, Table 1), although this may be partially caused by the motor deficit. To further investigate anxiety, we performed the elevated plus maze (EPM) test. The anxiety phenotype was seen in the young animals, but not in the older animals (Fig. 3D, Table 1). Remarkably, the WT animals tend to spend less time in the open arm at older age compared to younger age. In contrast, for the *Psip1* cKO mice the time in the open arm seems to be steady at younger and older age. The results of the EPM are thus in line with the results of the open field test at young age, but not at older age probably due to the aging effect in the WT group. The marble burying test is often used in the field of autism spectrum disorders to test compulsive-like and repetitive behavior [34, 56]. Others describe the burying as a defensive behavior against an aversive stimulus, such as the marbles suggesting that the behavior is anxiety-driven [34]. In contrast, in the field of RTT, marble burying is implemented to test for exploratory behavior, because mice are introduced to a novel environment [35]. The *Psip1* cKO mice show reduced burying, which can mean that they are less anxious, show less compulsive behavior or are less interested in exploring the novel environment (Fig. 3E-H, Table 1). Lastly, the Y maze test is commonly used to test for working and reference spatial memory. Both variants are based on the idea that mice have the innate curiosity to explore novel areas [57]. In the Y maze spontaneous test, both WT and cKO animals show a high percentage of spontaneous alternations at young age, while the cKO animals show a lower spontaneous alternations percentage at older age (Fig. 3I, Table 1). The reaction in the Y maze spontaneous test is based on the interaction between the prefrontal cortex and the hippocampus [57], which points towards a deficit in this interaction in aged cKO animals. In the second variant, the Y maze delayed test, we test for spatial reference memory by closing one of the arms during a training phase. Normally, the mouse should remember which arms it already explored during the training phase and should explore the novel arm. At young age, both groups seem to have no problems with distinguishing the novel arms from the familiar ones. However, at older age there seems to be no discrimination in recognizing the novel arm in cKO animals suggesting impairment in the hippocampal function (Fig. 3J, Table 1).

### The motor phenotype is not caused by a loss of dopaminergic neurons in SNpc

Since LEDGF is highly expressed in neurons, we verified if there was any neuronal loss in the *Psip1* cKO mice. We took advantage of an automated CNN-based model to detect NeuN staining across the full brain [32]. NeuN was quantified in both young and old animals, but no significant loss of neurons was detected (Fig. S3). Next, we performed specific double stainings in motor neurons in the spinal cord, Purkinje cells in the cerebellum and dopaminergic neurons in the SNpc. It is known that the Nestin^Cre^ conditional KO does not deplete the protein of interest in motor neurons or in Purkinje cells [53, 58] (Fig. 4A-B). Although a reduction of the LEDGF level was observed in dopaminergic neurons (Fig. 4C), no dopaminergic loss was evidenced at an age of 1 year (Fig. 4E). These results indicate that the observed motor phenotype cannot be attributed to overt neuronal loss throughout the brain nor to loss of SNpc dopaminergic neurons.

### Phenotypic similarities between the *Psip1* cKO model and mouse models depleted for LEDGF binding partners

We next compared the observed phenotype with that of mouse models for the binding partners of LEDGF. MeCP2 is interacting with both isoforms of LEDGF and loss-of-function mutations result in Rett syndrome (RTT) [7–10]. Several mouse models are available for RTT, including constitutive KO, conditional KO models or knock-ins of clinical mutants. In Table 2, we summarized the results from the available models. If we compare the *Psip1* cKO model with mouse models for RTT, an overlap is found with hypoactivity, less time on the rotarod, hindlimb clasping, deficits of spatial memory and the decrease in exploratory behavior (Table 2). Moreover, duplication of the *MECP2* gene results in MeCP2 duplication syndrome (MDS). The *Psip1* cKO model resembles the MDS mouse model in spending less time on the rotarod and the increased anxiety in the elevated plus maze (Table 2). However, most behavioral tests have not been performed in the MDS mouse models. For PogZ there is only one mouse model available, which is also a conditional KO in the central nervous system [18]. These mice also present deficits on the rotarod, in spatial memory and they also show less marble burying (Table 2). Lastly, there is also a *Mll1* KO mouse model available with a specific KO of *Mll1* in forebrain neurons [22, 25]. *Mll1* cKO mice show hypoactivity in the open field test, increased anxiety-like behavior and impairments in spatial memory (Table 2). Overall, the *Psip1* cKO phenotype resembles that of other animal models, but there is no animal model that gives a 100% overlap with our phenotype. The conditional and cell-specific nature of the LEDGF depletion also complicates the direct comparison.

**Table 2:** Phenotypes in different animal models

Phenotype	Test	*Psip1* cKO model (current study)	RTT model [15, 35, 59, 60]	MDS model [61–63]	*PogZ* KO model [18]	*Mll1* KO mouse model [22, 25]
Mouse model type		Conditional KO (Nestin^Cre^)	Constitutive KO Conditional KO ( e.g. Nestin^Cre^) Human pathogenic mutations	Overexpression	Conditional KO (Nestin^Cre^)	Conditional KO (CamK-Cre)
Growth delay	Body weight	No effect	Decreased	Decreased	Decreased	NT
Microcephaly	Brain weight	No effect	Decreased	No effect	Decreased	NT
General activity	Open field	Hypoactivity	Hypoactivity	NT	No effect	Hypoactivity
Motor coordination	Rotarod	Less time on the rod	Less time on the rod	Less time on the rod	Deficits	No effect
	Pole test	More time needed to turn	NT	NT	NT	NT
	Clasping	Present (hindlimb)	Present (hindlimb)	Present (forepaw)	NT	NT
Anxiety	Open field	More anxious	Less anxious	NT	No effect	More anxious
	Elevated Plus Maze	Heightened anxiety at young age	Less anxious	Heightened anxiety	No effect	Heightened anxiety
Spatial memory	Y maze spontaneous	Deficits at older age	Deficits (cross maze)	NT	Deficits (T maze)	Deficits (T maze)
	Y maze delayed	Deficits at older age	NT	NT	NT	NT
Exploratory behavior	Marble burying	Decreased	Decreased	NT	Decreased	NT

*NT* Not tested; *RTT* Rett syndrome; *MDS* MeCP2 duplication syndrome

### Cerebellar *Psip1* KO results in more and smaller MeCP2 condensates

For the analysis of LEDGF/p75 binding partners, we focused on the cerebellum and the hippocampus, as these 2 regions are possibly involved in the observed phenotype. Since the cerebellum has twofold more LEDGF/p75 than p52 and the hippocampus has fivefold more LEDGF/p52 than p75 these regions also allow to study the effect of the isoforms (Fig. 1A). We investigated 2 binding partners of LEDGF, MeCP2 and POGZ, in detail.

No difference in MeCP2 expression was observed in the cerebellum or hippocampus of *Psip1* cKO mice (Fig. 5B). In 2013, Becker et al. demonstrated that MeCP2 interacts with itself to form multimers [64]. In mouse cells, MeCP2 is present in chromocenters that are enriched for methylated satellite DNA repeats [65]. It has been proposed that the condensates represent liquid–liquid phase separation (LLPS) of MeCP2 due to multimerization of MeCP2 on chromatin [66]. MeCP2 clinical mutants seem defective for LLPS [66]. Previous research by our group revealed that LEDGF and MeCP2 do interfere with each other’s multimerization [9]. Lesire et al. corroborated the direct interaction between the PWWP-CR1 domain of LEDGF and the NID domain of MeCP2 and demonstrated that LEDGF depletion in mouse NIH3T3 cells increases the number of MeCP2E2 condensates [9]. Notably, MeCP2E2 has the highest expression in the cerebellum, more specifically in the granular layer, whereas MeCP2E1 is uniformly distributed throughout the brain [67]. Given the importance of MeCP2 condensates for MeCP2 function and the effect of LEDGF KD in cell culture, we quantified the number and size of MeCP2 condensates in the cerebellum and hippocampus. An increase in the number of smaller condensates in the cerebellum of *Psip1* cKO mice was observed (Fig. 5C and D). Similarly, there was a trend towards more and smaller MeCP2 condensates in the hippocampus of the *Psip1* cKO mice (Fig. 5E and F). The data are consistent with an increase in MeCP2 multimerization in the cerebellum after LEDGF depletion as seen in cell culture [9]. The functionality of MeCP2 condensates is still debated [40, 65] and no correlation between size and number of condensates and gene activation or repression by MeCP2 has been found. Likewise, the observed increase in the number of MeCP2 condensates may mimic MDS, whereas the smaller size of condensates may reflect RTT. In conclusion, LEDGF depletion affects MeCP2 condensates in mouse cerebellum, which could explain some of the behavioral changes and the resemblance to RTT or MDS (Table 2).

As for PogZ, no differential expression was found in the cerebellum or in the hippocampus of LEDGF depleted mouse brain (Fig. S4C). PogZ was expressed in all layers in both regions (Fig. S4A-B). Ibaraki et al. studied the PogZ expression during mouse brain development claiming the loss of PogZ expression in Purkinje cells from P30 onwards [68]. In contrast, we observed high expression of PogZ in the nucleus of Purkinje cells and in the nuclei of cells of the granular and molecular layer of the cerebellum at an age of 8 weeks and this both in WT and *Psip1* cKO mouse brain (Fig. S4B). Ibaraki et al. showed an enrichment of PogZ in the hippocampal neurons in the early developmental stage, but we observed enrichment of PogZ in hippocampal neurons even at older age (Fig. S4A) [68]. Finally, we compared the nuclear localization of PogZ in the cerebellum and hippocampus between WT and cKO mice. Since LEDGF/p75 acts as a molecular tether for its binding partners to chromatin, we hypothesized that the nuclear localization of PogZ could be affected in *Psip1* cKO mice. The *PogZ* mutations R997X, R1004X and E1043X that result in C-terminal truncations are known to relocalize PogZ to the cytoplasm [69]. Since LEDGF/p75 interacts with the transposase-derived DDE domain of PogZ [17], located in the C-terminal domain of PogZ, we hypothesized that the loss of LEDGF/p75 may also result in a cytoplasmic redistribution of PogZ. However, no redistribution of PogZ was evidenced (Fig. S4A-B).

### LEDGF acts as a major regulator of transcription in mouse brain

Isolated hippocampi and cerebella from WT and cKO mice were used to perform bulk RNA sequencing. In agreement with literature, LEDGF was found to act primarily as a transcriptional co-activator in mouse brain since we obtained more downregulated genes in our *Psip1* cKO mouse model [19, 46, 70–72] (Fig. 6A and B, Fig. S5A-B). Gene Set Enrichment Analysis (GSEA) indicates that gene sets involved in locomotor behavior are affected by *Psip1* KO in the cerebellum, in agreement with the observed motor phenotype in the open field and rotarod test (Fig. 2A–D, 6C). Moreover, *Psip1* KO impacts gene sets linked to GABAergic synaptic transmission (Fig. 6C, S7J), which is essential for the communication between Purkinje cells and deep cerebellar nuclei (DCN). It is known that there is no KO of *Psip1* obtained in the Purkinje cells of the cerebellum, however DCN process information from the Purkinje cells and generate the output of the cerebellum which is essential for motor coordination [73, 74]. Interestingly, alpha-synuclein encoded by the *Snca* gene proved to be downregulated in the cerebellum of the *Psip1* KO mouse (Fig. 7A, S8B, S9A-B). Since it is known that the expression of the *Snca* gene is downregulated by MeCP2 [75], this result may suggest that the observed increase in fine MeCP2 condensates increases its functionality. Whereas the role of alpha-synuclein in the pathogenesis of Parkinson’s disease and other synucleinopathies is associated by overexpression and aggregation, the protein is present in presynaptic terminals and the motor phenotype here may be related to a loss of function of its role in synaptic transmission [76, 77]. In conclusion, we hypothesize that the *Psip1* KO disturbs the synaptic transmission in the cerebellum and as a consequence impacts motor behavior.

MLL1 a binding partner of LEDGF/p75, is primarily studied for its role in Mixed Lineage Leukemia. LEDGF/p75 based tethering of MLL1 fusions to *Hox* genes is required for leukemogenesis but not for normal hematopoiesis [78]. MLL1 also plays a role in neurobiology, more precisely in neurogenesis, synaptic plasticity and working memory [22, 23]. MLL1 serves as the catalytic domain of Complex Proteins Associated with Set1 (COMPASS-like) complexes, which belong to the Trithorax group (TrxG) proteins [79]. These proteins are known for depositing a trimethyl group on histone 3 lysine 4 (H3K4), leading to gene activation. The binding between LEDGF/p75 and MLL1 is facilitated by menin, which acts as an adaptor [21]. The PWWP domain of LEDGF/p75 is important for tethering binding partners of LEDGF/p75, such as MLL1, to H3K36me2/3 marks in chromatin. Additionally, TrxG proteins have an antagonistic relationship with Polycomb group (PcG) proteins. PcG proteins form multimeric complexes on chromatin, resulting in repression of gene expression by methylating histone 3 lysine 27 (H3K27) [43, 79]. This repressive activity is dependent on the SET domain which is present in Enhancer Of Zeste Homolog 2 (Ezh2) [43]. Interestingly, 52% of differentially expressed cerebellar genes are regulated by the PRC2 complex, suggesting that the balance between TrxG and PcG protein complexes is disturbed upon depletion of LEDGF (Fig. 7A). Moreover, the downregulated genes, many synaptic genes, proved to be enriched for H3K27me3 histone marks, which is a hallmark of PRC2 activity. We hypothesize that LEDGF/p75 is involved in the recruitment of the TrxG protein MLL1 complex to chromatin and that in its absence the MLL1 complex dissociates from the chromatin tilting the balance towards more PRC2 complex activity and more gene repression.

**Fig. 8:**
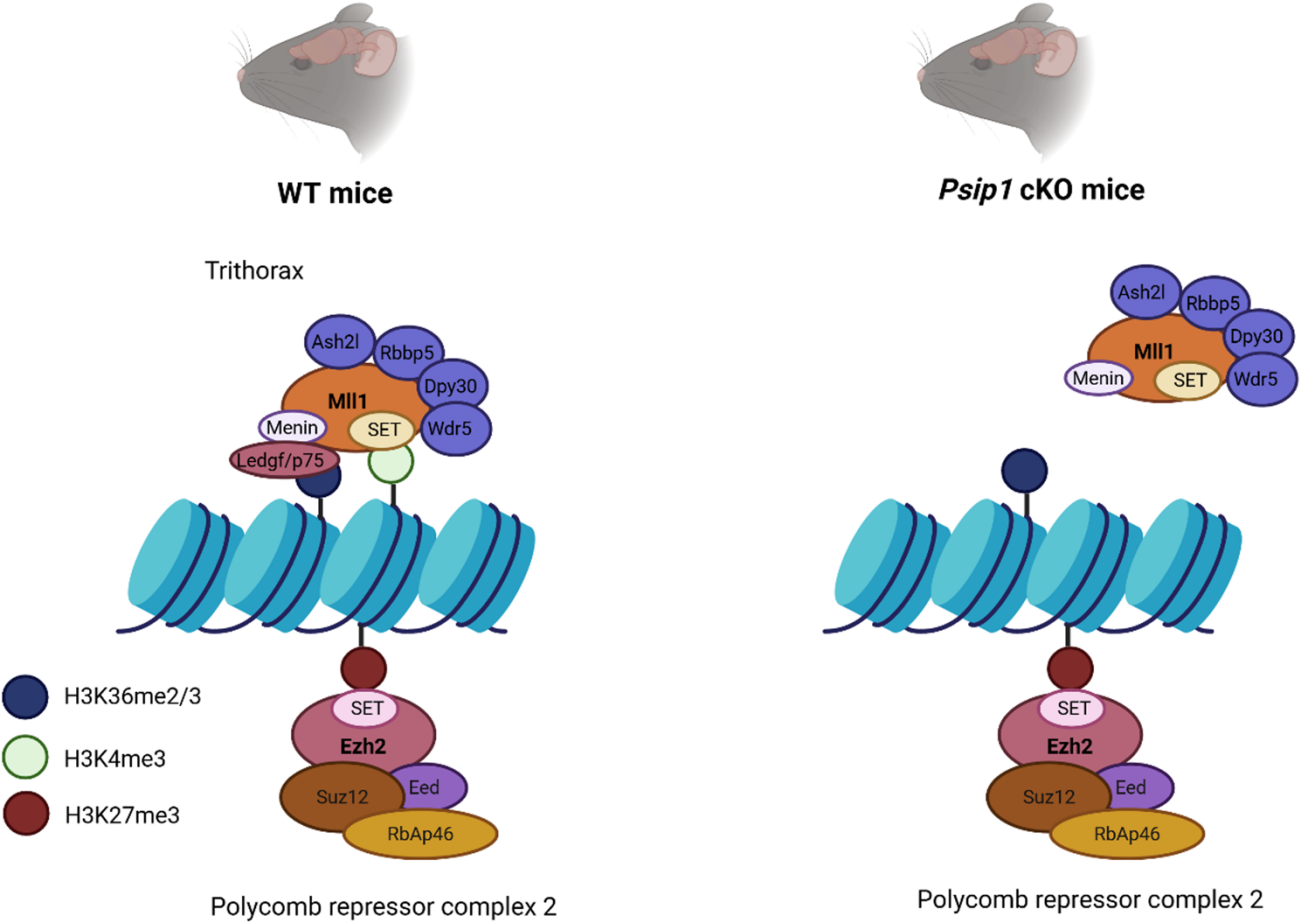
Model for LEDGF mediated control of gene expression. In WT mice transcription is regulated by keeping the trithorax and polycomb repressor complex 2 (PRC2) in balance. LEDGF/p75 act as a molecular tether to guide the trithorax system to active genes. However, in *Psip1* cKO mice, LEDGF/p75 is no longer present resulting in the loss of binding of the trithorax system to chromatin. As a result, the PRC2 complex is more active resulting in more downregulated genes. Ezh2 = enhancer of zeste homolog 2, Eed = embryonic ectoderm development, Suz12 = suppressor of zeste 12, RbAp46 = Histone binding protein, SET = Sur3-9 Enhancer-of-zeste, Ash2l = ASH2 Like, Rbbp5 = Retinoblastoma-Binding Protein 5, Dpy30 = dpy-30 histone methyltransferase complex regulatory subunit, Wdr5 = WD Repeat Domain 5.

To conclude, we provide a comprehensive analysis of the phenotypic and molecular impact of conditional LEDGF depletion in mouse brain. This study highlights the important role of LEDGF in brain function mediated by binding partners MeCP2 and MLL1. The impact of LEDGF depletion on MeCP2 condensates may explain part of the behavioral phenotype. LEDGF/p75-based tethering of the MLL1 complex to chromatin activates cerebellar genes (Fig. 8). LEDGF/p75 thereby controls the balance between the TrxG and PcG protein complexes, resulting in activation or repression of genes, respectively. The depletion of LEDGF tilts the balance between PcG and TrxG protein complexes towards the PRC2 complex and downregulation of gene expression (Fig. 8).

## Data Availability

The datasets used and/or analysed during the current study are available from the corresponding author on reasonable request.
